# Kalpana Balakrishnan: the power of data to drive positive change

**DOI:** 10.2471/BLT.19.030219

**Published:** 2019-02-01

## Abstract

Gary Humphreys talks to Kalpana Balakrishnan about her background in biophysics and how science has informed her public health work.

Q: You grew up in India, but moved to the USA in 1984 where you obtained a doctoral degree in biophysics at Johns Hopkins University. How has that beginning in science influenced your work in public health?

A: Well, I think this gave me the foundation to approach things on the basis of first principles. I spent my doctoral years training in neurotoxicology, looking at how neurons respond to different kinds of signals, how they process the signals, and what affects signal transduction. So it was a really hardcore mechanistic way of trying to figure out how things work at the cellular level, a paradigm I continue to apply in the larger realm of public health.

Q: How did you get from there to public health?

A: The thing that really triggered the transition was exposure to new areas of study during my post-doctoral years. One of my advisors at Johns Hopkins encouraged me to take a couple of courses on population dynamics and health economics just to make me appreciate how I might consider applying my mechanistic knowledge. It was not easy, partly because it was so far away from my laboratory-based skills, but it turned out that I found a perfect niche.

Q: Which was?

A: Essentially applying my knowledge of physiology and toxicology and looking at the mechanisms of normal and abnormal response at the cellular level based on the dose of external stimuli. Morton Corn, Patrick Breysse and Peter Lees – my advisors at Johns Hopkins – trained me on the fundamentals of how you can measure things in the environment and assess health risks. I was looking at occupational exposures to asbestos, cadmium, beryllium and nickel. The context of the work was industrial, but the methods were extendable to the general environment.

Q: You returned to India in 1996. Were you able to apply those skills when you got home?

A: Fortunately, yes. I went to several government departments to talk about my work, and I ended up at here at the Sri Ramachandra Medical College & Research Institute (now Sri Ramachandra Institute of Higher Education and Research) in Chennai. I am a researcher at heart and I really wanted to kick-start research into occupational exposures within the medical university.

Q: What was your first project?

A: I started by looking at health risks from exposure to chromium. I was fortunate enough to get access to some industrial units handling chromium. That was a defining moment for me. I went to this factory and I saw these workers who were just drenched in chromium. I came back home and just sat and cried. I was thinking how futile it was to produce another study on chromium toxicity. I said to myself I'm sure I can understand a bit more by spending another 30 years on exactly how chromium affects human tissue, but who cares? That guy in that factory is dying!

Q: So that was your induction into a public health way of thinking.

A: It certainly was. I knew I didn’t just want to showcase these problems. I wanted to do something about them. Which I did, by bringing evidence to the attention of large and small industries. Not just on chromium, but on a wide range of harmful environmental factors in a variety of sectors, ranging from leather and textiles to power, chemical manufacturing and cars.

“… spending another 30 years on exactly how chromium affects human tissue, but who cares? That guy in that factory is dying!”

Q: How did industry view this?

A: With misgivings initially. The manufacturing industry at that point was so skeptical of these kinds of assessments because they thought they would appear in the papers the next day. But they let us in to do the assessments and make the recommendations. It was all about finding solutions, suggestions that they could consider internally. Finger-pointing without proposing feasible solutions is not constructive.

Q: Do you feel you were able to make a difference even though the industries you were looking at were self-regulating?

A: I don't think we can possibly make a dent in the magnitude of the problem they have, but we have created a movement, and many industry sectors have benefited from our assessments and guidance on solutions. 

Q: So at what point did you start working on the health impact of indoor air pollution? Did it come out of the work you were doing in industry?

A: Yes it did. Initially we were focused on the occupational environment, but then we started thinking about population level exposures. It was at this time that I met Professor Kirk Smith of UC Berkley, who is one of the pioneers of air pollution research, and, incidentally, also a physicist by training. In 1988 we started going to communities, some rural, some peri-urban, looking at ambient pollution monitors, but we felt that they were only getting part of the picture because no measurement was going on in people’s homes. So I proposed using the instruments that we used to measure workers’ exposures to assess women’s exposures and child exposures to household air pollution. The big breakthrough came when in 2000 when we were given a four months contract to measure levels of particulate matter in 400 biomass-using households.

Q: How did you make the measurements?

A: We used air sampling pumps that mimic the deposition of particulate matter into the lungs. We presented the results of the 400 household measurements in the first WHO consultation in 2002. That same year WHO invited me to present the epidemiological evidence on the risks from household, solid fuel use at the World Health Assembly. WHO had put together an assessment comparing the disease burden attributable to different health risk factors such as salt intake, tobacco use etc. Lo and behold in South Asia and India, household air pollution ended up ranking amongst the top 10 risk factors. This came as a complete surprise. That comparative risk assessment made a huge difference to awareness of the issue and started people thinking about what to do in response.

“There is nothing more powerful than data if you want to bring about change.”

Q: You also carried out a study looking at the impact of a pregnant mother’s exposure to air pollution on her baby’s birthweight. 

A: That’s right. We examined whether exposure to fine particulate matter (PM_2.5_) during pregnancy were associated with lower birthweight. The study provided some of the first quantitative effects estimates for linking rural-urban PM_2.5_ exposures and birthweight in India. 

Q: So the evidence of harm was steadily accumulating. What came out of it in terms of a response?

A: Initially we didn’t quite know how to address the issue because there was no access to clean energy in India. The only thing that was sort of working were these improved cooking stoves. In the field, however, we’d see these stoves just lying in the corner; nobody was using them because they couldn’t really hold the pans that a traditional stove would take, and would break down most of the time. So after studying the evidence for a number of years we said, “Let’s reframe our thinking. Let’s stop trying to improve the way we burn biomass and focus on clean fuels.” By 2014 when the household air pollution guidelines for WHO were being formulated, there was enough evidence to say that liquid propane gas (LPG) is likely the only technology that, when scaled, can consistently meet the minimum emission and exposure targets set by the WHO guidelines. We knew there was a lot of demand for LPG in India, and we knew also that 40% of the world’s population were already using clean fuels. So it seemed logical and ethical to look for ways of making this available to the other 60%. 

Q: Is LPG available everywhere in India?

A: Not everywhere, and as you might expect, distribution problems and access problems arise more in rural areas. LPG distribution is highly regulated in India and is also subsidized by the government. The oil marketing companies bottle the LPG and supply registered, authorized distributors who sell to consumers. There have been a number of consultations about expanding access by enlarging LPG distributor networks. They want to scale distribution to a point where anybody who wants to use LPG can. Currently coverage is around 70-80% in rural areas; the government wants to get to 95% by 2020.

Q: Do you think they will reach that target? 

A: Well, thus far, the programme has exceeded its targets. So it seems very likely.

Q: What about alternatives to LPG, such as electricity, perhaps generated through solar power?

A: I think we have the ability to develop renewable electricity in India on a huge scale. If the investment in infrastructure is made why not? But right now, LPG is perfect. 

Q: What about the future for yourself? 

A: Well first I would say that, in my opinion, nobody has the bandwidth to do high level policy and hard-core field research at the same time. And for me the choice is simple. I just want to be a scientist! If it were not for the encouragement provided by WHO and some other reputed bodies, I would never stand up and speak my mind about what should or should not be done. I would much rather be in the lab, or the field and let my data do the talking. I believe that it is by engaging in ground-based research that I can contribute the most for this issue. Because there is nothing more powerful than data if you want to bring about change. Unfortunately, not many of us in developing countries are generating solid data. 

